# The cost-effectiveness of second line diabetes medication added to standard therapy in preventing type 2 diabetes related nephropathy in Germany

**DOI:** 10.1186/s12962-026-00801-5

**Published:** 2026-07-27

**Authors:** Hanni Hille, Wendelin Schramm, Ahmed Bounekkar

**Affiliations:** 1 GECKO Institute, Hochschule Heilbronn, Max-Planck Straße 39, 74081 Heilbronn, Germany; 2 ERIC Laboratory, Claude Bernard University Lyon 1, Lyon, France

**Keywords:** Type 2 diabetes mellitus, Diabetic nephropathy, Medical economics, Cost-effectiveness analysis, Markov chains, Tirzepatide, Semaglutide, Empagliflozin

## Abstract

**Background:**

Type 2 diabetes and its associated complications like nephropathy impose a serious burden on health systems. Several blood glucose-lowering medications have shown to be likely to provide nephroprotective properties, delaying or preventing the onset of kidney damage. Kidney-specific health-economic evaluations, comparing different agents, remain rare for Germany.

**Methods:**

A Markov-microsimulation model was developed to obtain the perspective of the German statutory health system regarding cost-effectiveness. The model consists of five health states representing varying degrees of kidney damage with transition probabilities, utilities and costs being taken from published literature. Patient age was set to 62.8 years in the primary cohort; an additional analysis scenario was created with a start age of 50 years. The model outcomes life expectancy, quality adjusted life years, costs and net monetary benefit were discounted at 3% at a willingness-to-pay threshold of 100,000€ per quality adjusted life year gained.

**Results:**

Quality adjusted life years increased from 10.28 years in standard care to 10.48 years with Empagliflozin, 10.58 years with Semaglutide and 10.75 years with Tirzepatide all added on top of standard care. Life expectancy increased from 13.36 years in the base case to 13.66 years, 13.76 years and 14.00 years with Empagliflozin, Semaglutide and Tirzepatide respectively. Total direct cost amounted to 66,343.64€ in the base case, 79,767.42€ with Empagliflozin, 84,328.59€ with Semaglutide and to 155,992.59€ with Tirzepatide. The net monetary benefit was calculated to be 961,445.98€ in the base case, 968,406.95€, 973,647.74€ and 919,972.95€ with Empagliflozin, Semaglutide and Tirzepatide respectively. Multiple one-way sensitivity analyses were performed. The most influencing factors on the cost-effectiveness were found to be quality of life in the type 2 diabetes state and intervention cost.

**Conclusion:**

In this study, the application of second-line glucose-lowering medication in type 2 diabetes patients led to relevant medical improvements regarding kidney damage at higher expenditures. At the chosen willingness-to-pay threshold Empagliflozin and Semaglutide remained cost-effective in all scenarios. Patient age and sex did not change the health-economic implications.

**Clinical trial number:**

Not applicable.

**Supplementary Information:**

The online version contains supplementary material available at 10.1186/s12962-026-00801-5.

## Introduction

### Background

Type 2 diabetes (T2D) is one of the most prevalent metabolic disorders on a global scale [1]. In Germany approximately 9.60% of the adult population is diagnosed with T2D as of 2023 [2]. An increase of 1.6% in the adult T2D prevalence was predicted by Ogurtsova et al. [3] for the years between 2015 and 2040. In Germany T2D represents a serious burden on the German public health system by not only being a major challenge for public health but also being one of the most expensive chronic diseases [4]. The financial burden is driven by complications associated with T2D such as cardiovascular disease, retinopathy and amputations.

Diabetic nephropathy is one of the severe complications associated with T2D [5]. It can be characterized by progressive albuminuria and a decrease in estimated glomerular filtration rate (eGFR). Progressions from microalbuminuria to macroalbuminuria can eventually reach renal failure often requiring dialysis or kidney transplantation. This puts a critical burden on health and quality of life of patients as well as for the statutory health system.

An important clinical and economical question therefore is how to delay or avoid the onset and progression of diabetic nephropathy. Certain blood glucose lowering medication such as Semaglutide [6] and Empagliflozin [7] have proven to be likely to provide nephroprotective properties beyond their glycaemic effects. Early application of such medications could preserve renal function leading to improved quality of life and major reductions in healthcare expenditures.

A well-established approach for evaluating long-term cost-effectiveness is health-economic modelling especially for chronic diseases [8]. Patient cohorts can be simulated through predefined health states over a specific time horizon. These models capture costs and clinical outcomes beyond the possibilities of typical clinical trials providing an important decision-making tool for healthcare systems.

The cost-effectiveness of several second-line antidiabetic drugs has been assessed in international studies before, but there is a lack of German models focusing on renal outcomes specifically [9, 10]. There is limited evidence comparing a wide range of glucose-lowering agents considering their effects on nephropathy progression while assessing long-term costs within the German statutory health insurance context.

### Research objective

This exploratory cost-effectiveness study followed the objective to simulate potential effects and costs for the use of additional T2D medication to prevent nephropathy from the perspective of the German statutory health system using a health economic disease model. Thus, the meaning of what is publicly available regarding medical, economic and health-economic values was assessed. The following research question was to be answered:

Can the addition of T2D interventions like Tirzepatide, Semaglutide and Empagliflozin to standard blood glucose medication effectively prevent the development or at least slow the progression of renal disease while being affordable for the German statutory health system?

## Methods

The Consolidated Health Economic Evaluation Reporting Standard (CHEERS) is followed in this study.

### Study populations

In this study, patient characteristics reported in the 2015 ACCORD study [11] were used to build the study cohort. No original patient data was gathered which is why an ethics approval was not needed. Table 1 provides an overview of the patient characteristics used in this model study. An alternative cohort was defined with a default start age of 50 years to analyse life expectancy related age effects on the health-economic outcomes.

**Table 1:** Patient characteristics

Patient characteristic	Input value
Age	62.8 years / 50 years
Gender	Male / Female
Anticoagulants	No
Anti-hypertensive drugs	No
BMI	32.2
Blood pressure drugs	No
CVD history	No
HbA1c	8.3%
HDL	41.8 mg/dL
Black ethnicity	No
Hispanic ethnicity	No
Oral T2D drugs	Yes
Systolic blood pressure	136.5 mmHg
Serum creatinine	0.9 mg/dL
Smoker	No
Total cholesterol	183.20 mg/dL
Urin albumin creatinine ratio	99.2

### Setting and location

Cost data for Germany, including state and medication cost, were gathered from a reliable cost of illness study by Kähm et al. [12]. Risk equations, transition probabilities and treatment effects were taken from US American studies due to no German literature being available at the time of this study. With similar baseline patient characteristics these studies were assumed to be applicable to a German context. Table 5 summarizes all model input parameters along with their references.

### Comparators

This exploratory cost-effectiveness study investigates four interventions. As the base case, standard T2D treatment was chosen which in Germany, based on treatment guidelines, includes Metformin [13]. All three comparators were applied additionally to standard T2D treatment, as second-line treatment. Second-line treatment in Germany is prescribed in situation of existing co-morbidities or when blood-glucose thresholds cannot be achieved with first-line treatment alone. For GLP-1 receptor agonists, Tirzepatide was chosen at a dosage of 15 mg per week. Semaglutide was chosen at 1 mg per week for the treatment of diabetes, as approved of by German regulations [14]. For comparison against a different working mechanism, the SGLT-2 inhibitor Empagliflozin was chosen at a dosage of 25 mg per day.

Treatment effects for HbA1c are considered in percent, HDL and total cholesterol changes are considered in mg/dL. Literature values for HDL and total cholesterol reporting in mmol/L were converted as follows: mg/dL = mmol/L * 38.67.

### Health economic perspective

This study obtains the perspective of the German statutory health system. Only direct medical care and intervention costs were considered, leaving out indirect medical costs and out-of-pocket expenses made by patients. Left out were prior patient costs, additional illnesses and other treatments.

### Time horizon

The model was developed to simulate 60 annual cycles capturing long-term treatment effects and respective costs.

### Discount rate

All outcomes were evaluated using a 3% discount rate, in accordance with the 2023 recommendations of the German Institute for Quality and Efficiency in Health Care (IQWIG) [15].

### Selection of outcomes

For the valuation of clinical effectiveness, life expectancy (LE) and quality adjusted life years (QALY) were chosen as main health outcomes.

Economic effectiveness was assessed using total direct cost over the remaining patient lifespan including stage cost and annual medication expenses.

Health economic outcomes are presented as the incremental cost utility ratio (ICUR) of quality adjusted life years gained with the corresponding net monetary benefit (NMB).

### Measurements of outcomes

Due to the model operating on annual cycles, costs and utilities were assigned on an annual basis as well. QALYs were derived by multiplying the duration spent in each health state by the utility weight associated with the state. Total direct cost and QALYs were summed over the remaining lifetime.

The ICUR was obtained for economic evaluation by comparing incremental total cost and QALY with those in the base case scenario. A willingness-to-pay (WTP) threshold of 100,000€ per QALY gained was used as commonly applied in CE-analyses [16].

For each calculation, the NMB was computed as QALYs multiplied by the WTP threshold minus total cost enabling a non-incremental comparison and ranking of comparators.

### Valuation of outcomes and resources

All input parameters were taken from recent scientific literature and applied to a patient cohort with a baseline age of 62.8 years.

### Currency, price date and conversion

For all comparators, costs are estimated in 2025 Euros. Stage costs in the five health states and additional costs for the respective intervention were considered. Stage costs were based on the insights by Kähm et al. [12] and adapted to the reference year 2023, the last year of complete records of the health sector specific expenditures from the Federal Statistical Office of Germany at the time of the model computations. All stage costs are listed in Table 2.

**Table 2:** Stage and medication costs for Germany in the reference year of 2023

Cost category	Stages/ Events	Annual cost (€)	Source
Stage & event cost	T2D	3,772.48	[12]
	First year microalbuminuria	8,615.75	[12]
	Following microalbuminuria	7,109.76	[12]
	First year macroalbuminuria	8,615.75	[12]
	Following macroalbuminuria	7,109.76	[12]
	First year renal disease	44,213.85	[12]
	Following renal disease	31,911.91	[12]

Additionally, medication cost was added onto the respective stage cost per year. For Semaglutide 1 mg 1,283.75€ was added, for Tirzepatide 15 mg 6,261.84€ and for Empagliflozin 25 mg 694.92€ respectively. All cost data was taken from the German online pharmacy ShopApotheke [17].

Medication costs were taken from a German pharmacy price catalogue [17] in 2025 and adjusted to an annual price based on the respective dosage.

### Rationale and description of the model

A blueprint for the model was derived from the previously existing nephropathy model from the PROSIT disease modelling framework [18], which in combination with the other PROSIT complication models successfully participated in the 2018 Mt. Hood Diabetes Network Challenge [19]. The Markov Monte Carlo patient simulation model for this study was developed using the software TreeAge Healthcare Pro 2025 R1.1 [20] applying Markov calculations and microsimulation. The Markov model lets individuals occupy the given set of health states with time being divided into cycles. In each cycle, movement of individuals between states is decided by transition probability matrixes. Costs and utilities are calculated by weighing time spend in a state according to its respective costs and utilities over all cycles.

**Fig. 1:**
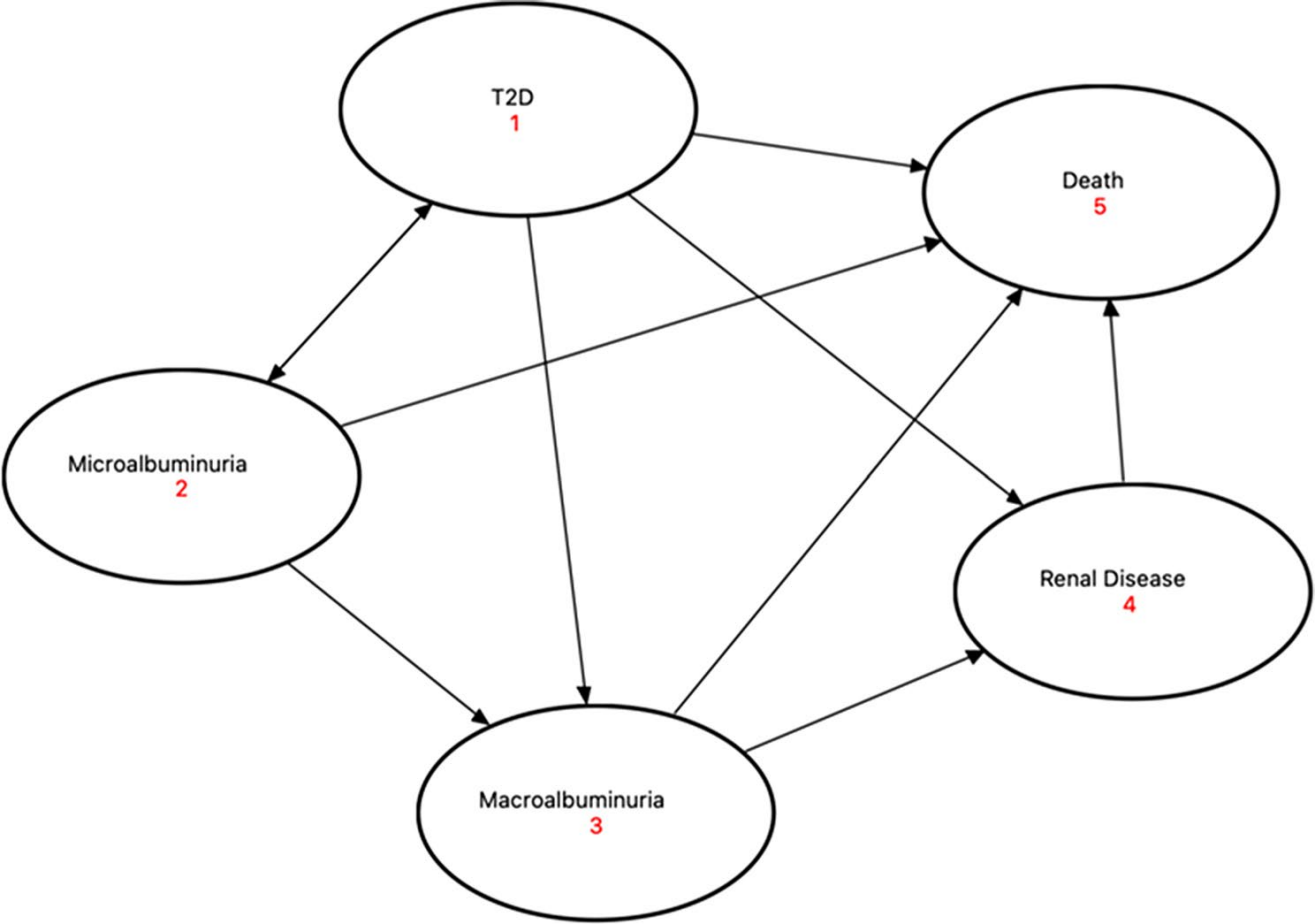
State transition diagram

Five health states are included in the model as shown in Fig. 1, namely type 2 diabetes (T2D), microalbuminuria, macroalbuminuria, renal disease and death. Health states were differentiated by albumin in the urine and severity of kidney damage. Regression is only possible from microalbuminuria back to T2D. Furthermore, renal disease cannot develop directly from microalbuminuria.

### Analytics and assumptions

If a comparator was not to be considered cost-effective at its list price with the chosen WTP threshold, a price threshold analysis was performed. The unit cost was varied until its incremental cost-effectiveness ratio equalled the willingness-to-pay threshold, thereby identifying the maximum price at which it would be considered cost-effective.

Assumptions had to be made during model development.

All risk equations, transition probabilities, quality of life (QoL) values and treatment effects used in this model were taken from published scientific literature. All numerical values for QoL, transition probabilities, mortality values and discount rates are listed and referenced.

General mortality in the T2D state was calculated by subtracting the number of deaths caused by nephropathy from the number of total deaths in Germany per age group and sex in order to avoid double counting. Data was taken from the German federal statistical office (Statistisches Bundesamt) [21].

Duration of diabetes was not directly used in this study’s risk equations. The average age for diabetes manifestation in Germany is typically lower than the patient age in this study which is why it can be assumed that the patients used are not newly diagnosed. Patients were assumed to be diagnosed with T2D more than five years ago.

Furthermore, it is assumed that medical interventions like Tirzepatide, Semaglutide and Empagliflozin provide renal protection leading to slowed progression of T2D related kidney damage. Base case transition probabilities from T2D to any nephropathic state were altered indirectly through intervention effects on patient characteristics as listed in Table 3. Hazard ratios (HR) representing organ protective effects were added for all other transitions in the model. Table 4 shows which patient characteristics influence the transition probability in the model and in which comparator scenario.

**Table 3:** Influence of T2D medication on baseline patient characteristics

	Base case	Semaglutide 1 mg/ week	Tirzepatide 15 mg/ week	Empagliflozin 25 mg/ day
HbA1c (%)	0.00	-1.50 [22]	-2.30 [23]	-0.36 [24]
HDL (mg/dL)	0.00	2.51 [25]	4.80 [23]	0.00 [26]
Total cholesterol (mg/dL)	0.00	-4.02 [25]	-10.4 [23]	0.00 [26]

**Table 4:** Influence of patient characteristic on respective transition

From state	T2D				Microalbuminuria			Macroalbuminuria		Renal disease
To state	Micro-albuminuria	Macro-albuminuria	Renal disease	Death	T2D	Macroalbuminuria	Death	Renal disease	Death	Death
Initial Age	x	x	x	x	x		x		x	x
Gender	x	x	x	x	x		x		x	
BMI										
Anticoagulants	x	x	x							
Anti-hypertensive drugs	x	x	x		x					
Oral T2D drugs	x	x	x							
CVD history	x	x	x							
HbA1c	x●■★	x●■★	x●■★		x					
HDL	x●■★	x●■★	x●■★							
Black ethnicity	x	x	x							
Hispanic ethnicity	x	x	x							
SBP	x	x	x							
Serum creatinine	x	x	x							
Smoker	x	x	x							
Total cholesterol	x●■★	x●■★	x●■★							
Urin albumin creatinine ratio		x	x							
HR intervention specific						●■★		●■★		●■★

X Standard, ● = Tirzepatide, ■ = Semaglutide, ★ = Empagliflozin

### Characterizing heterogeneity

Due to sex related differences in health risks, like different mortality values, men and women were simulated separately to prevent shifting the sex shares in the cohort.

Additionally, a second computation was performed for both sexes with the start age lowered to 50 years to investigate its influence on health economic outcomes.

### Characterizing distributional effects

This study uses reported data without distribution information according to the underlying publications. No further differentiation was made regarding subgroups in the main analysis.

### Characterizing uncertainty

For technical validation, expected values were calculated to assure valid parameter sets and to identify technical faults such as branch probabilities above 100%.

The final version of the model underwent internal validation through one-way sensitivity analysis (SA) examining the effect of each numerical value on the incremental NMB. Costs, transition probabilities and QoL values were varied by ± 10%.

### Approach to engagement with patients and others affected by the study

No direct involvement of patients or healthcare professionals was performed, as this study relied on previously published person-level data. Under these conditions the identification of real persons is not possible within the scope of this study.

## Results

### Study parameters

The aggregation of model parameters was described before. Table 5 summarises all values along with their references.

Different hazard ratios for organ-protective effects, depending on the respective medical intervention, were implemented to all progressions amongst nephropathic states. For Tirzepatide a HR of 0.58 [27], for Semaglutide 0.79 [28] and for Empagliflozin a HR of 0.61 [29] was used.

Quality of life estimates were derived from the published review by Beaudet et al. [30] which reports utilities originally presented by Bagust et al. [31]. Age and sex specific decrements in health-related quality of life were applied to these baseline utilities.

**Table 5:** Values for quality of life, transition probabilities and mortality

Parameter	Value	Comment
Start age in years	62.8 years / 50 years	
Quality of life in T2D	Value dependent on age and sex	Value according to [30, 31]
Quality of life in microalbuminuria	Value dependent on age and sex	Value according to [30, 31]
Quality of life in macroalbuminuria	Value dependent on age and sex	Value according to [30, 31]
Quality of life in renal disease	Value dependent on age and sex	Value according to [30, 31]
Quality of life in death	0	
General mortality without nephropathy	Table according to age and sex	Based on values from German federal statistical office for the year 2023 (Death statistic 23211-0002 and 12411-0006)
Transition probability from T2D to microalbuminuria	Risk equation * intervention specific HR	Risk equation by Basu et al. [32]
Transition probability from T2D to macroalbuminuria	Risk equation * intervention specific HR	Risk equation by Basu et al. [32]
Transition probability from T2D to renal disease	Risk equation * intervention specific HR	Risk equation by Basu et al. [32]
Transition probability from T2D to death	Formula dependant on age and sex	See general mortality
Transition probability from microalbuminuria to T2D	Formula	Formula based on Newman et al. [33]
Transition probability from microalbuminuria to macroalbuminuria	0,028 * intervention specific HR	Transition probability by Adler et al. [34]
Transition probability from microalbuminuria to death	Formula dependant on age and sex	Formula based on Newman et al. [33]
Transition probability from macroalbuminuria to renal disease	0,023 * intervention specific HR	Transition probability by Adler et al. [34]
Transition probability from macroalbuminuria to death	Formula dependant on age and sex	Formula based on Valmadrid et al. [35]
Transition probability from renal disease to death	Formula dependant on sex	Formula based on Van Dijk et al. [36]

### Medical outcomes

Women live approximately 4.5 years longer than men in the general German population. A sex difference between 1.78 and 1.98 years was calculated between men and women.

All interventions are effective to prevent kidney related complications against the standard of care. This leads to a decrease in development of microalbuminuria, macroalbuminuria and renal disease in all scenarios. In the base case 31.46% of patients developed microalbuminuria from T2D whereas with Empagliflozin 31.26%, with Semaglutide 27.73% and with Tirzepatide 25.39% respectively. From microalbuminuria in the base case 4.12% of patients developed macroalbuminuria, with Empagliflozin, Semaglutide and Tirzepatide 2.26%, 2.37% and 1.42% respectively. Patients with macroalbuminuria developed end stage renal disease in 2.82% of cases in the base case whereas with Empagliflozin, Semaglutide and Tirzepatide 1.69%, 1.96% and 1.33% respectively.

**Fig. 2:**
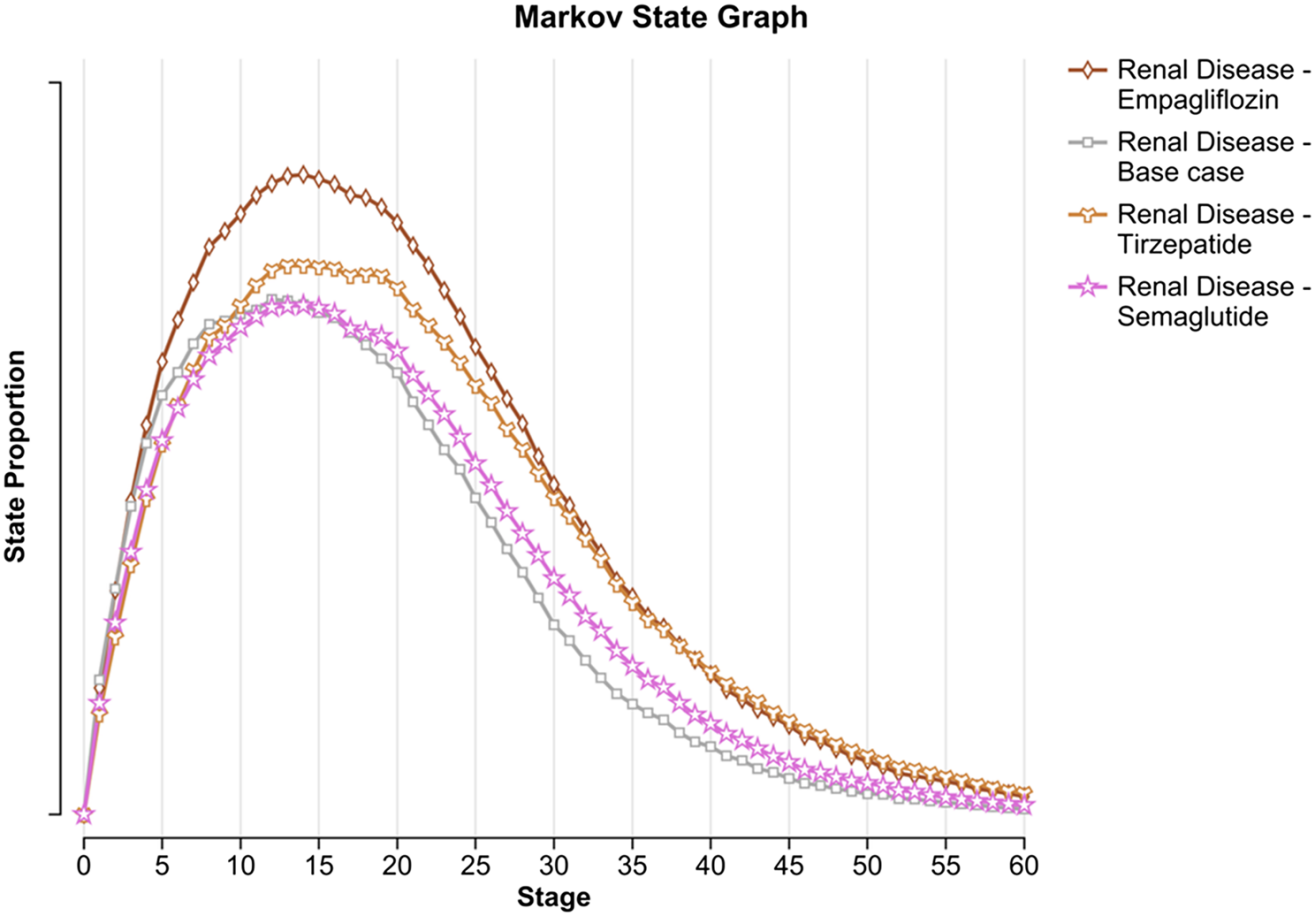
Annual incidence of renal disease in 62.8-year-old male patients at the start by intervention

Annual incidence of renal failure per comparator is depicted in Fig. 2.

### Economic outcomes

Total direct costs in the base case scenario with standard T2D medication were calculated with 66,343.64€ for men and 78,662.94€ for women on average.

Empagliflozin represents the most affordable treatment option with a total direct cost of 79,767.42€ for men and 93,556.45€ for women on average. All other outcomes are illustrated in Table 6.

### HE outcomes

As depicted in Table 6 Empagliflozin and Semaglutide are considered cost-effective with their ICUR laying below the WTP of 100,000€ per QALY gained.

All medical, economic and health economic results are summarised in Table 6.

**Table 6:** Main health economic outcomes before each comparator in the 62.8-year-old cohort separated by men and women

Strategy	Costs [€] male (Monte Carlo SE) female (Monte Carlo SE)	Incr. Costs [€]	Life expectancy [LE]	Incr. Life expectancy [LE]	Utilities [QALY]	Incr. Effectiveness [QALY]	ICUR [IC/IQALY gained]	NMB based on QoL [€]
Standard of care (Base case)	**66**,**343.64** *(66*,*078.08-66*,*609.20)* **78**,**662.94** *(78*,*381.87-78*,*944.01)*	-	**13.36** **15.14**	-	**10.28** *(10.25–10.30)* **10.16** *(10.14–10.18)*	-	-	**961**,**445.98** *(958*,*944.56–963*,*947.40)* **937**,**323.17** *(935*,*070.78–939*,*575.55)*
Empagliflozin	**79**,**767.42** *(79*,*394.63-80*,*140.21)* **93**,**556.45** *(93*,*171.44-93*,*941.46)*	13,423.78 14,893.51	**13.66** **15.53**	0.30 0.39	**10.48** *(10.46–10.51)* **10.40** *(10.37–10.42)*	0.20 0.24	67.118,90 62,056.29	**968**,**406.95** *(965*,*920.49–970*,*893.41)* **946**,**282.98** *(944*,*946.98–948*,*519.89)*
Semaglutide	**84**,**328.59** *(84*,*009.65-84*,*647.54)* **98**,**795.48** *(98*,*463.07-99*,*127.90)*	17,984.95 20,132.54	**13.76** **15.66**	0.40 0.52	**10.58** *(10.55–10.61)* **10.51** *(10.48–10.53)*	0.30 0.35	59,949.83 57,521.54	**973**,**647.74** *(971*,*143.20–976*,*152.27)* **952**,**016.42** *(949*,*765.57–954*,*267.27)*
Tirzepatide	**155**,**992.59** *(155*,*499.34–156*,*485.84)* **180**,**312**,**45** *(179*,*807.29–180*,*817.60)*	89,648.95 101,649.51	**14.00** **15.98**	0.64 0.84	**10.75** *(10.73–10.78)* **10.71** *(10.69–10.53)*	0.47 0.55	190,742.45 184,817.29	**919**,**972.95** *(916*,*924.77–921*,*621.14)* **890**,**809.07** *(888*,*719.71–892*,*898.43)*

### Effect of uncertainty

In all SAs patient age was found to be amongst the four most influential factor. As this was to be anticipated, a second analytical scenario was computed with a lower patient age cohort to test the model’s robustness against the age as source of uncertainty.

QALY values and life expectancy are expectantly higher when observing younger patients even though health economic implications remained unchanged with Empagliflozin and Semaglutide being cost-effective. This indicates that the health economic outcomes are not influenced by patient age.

All medical, economic and health economic results for the alternative simulation scenario are summarised in Table 7.

**Table 7:** Main health economic outcomes for each comparator in the 50-year-old cohort separated by men and women

Strategy	Costs [€] male (Monte Carlo SE) female (Monte Carlo SE)	Incr. Costs [€]	Life expectancy [LE]	Incr. Life expectancy [LE]	Utilities [QALY]	Incr. Effectiveness [QALY]	ICUR [IC/IQALY gained]	NMB based on QoL [€]
Standard of care (Base case)	**90**,**443.29** *(90*,*157.36-90*,*729.22)* **102**,**002.08** *(101*,*710.75–102*,*293.42)*	-	**17.44** **18.86**	-	**13.26** *(13.23–13.29)* **12.49** *(12.47–12.52)*	-	-	**1**,**235**,**438.27** *(1*,*232*,*781.55-1*,*238*,*094.70)* **1**,**147**,**332.19** *(1*,*145*,*008.35-1*,*149*,*656.02)*
Empagliflozin	**109**,**053.32** *(108*,*646.51–109*,*460.13)* **121**,**934.93** *(121*,*528.16–122*,*341.70)*	18,610.03 19,932.85	**17.90** **19.43**	0.46 0.57	**13.57** *(13.55–13.60)* **12.84** *(12.81–12.86)*	0.31 0.35	60,032.35 56,951.00	**1**,**248**,**272.33** *(1*,*245*,*681.78-1*,*250*,*862.88)* **1**,**161**,**665.04** *(1*,*159*,*410.38-1*,*163*,*919.70)*
Semaglutide	**113**,**860.48** *(113*,*522.27–114*,*198.70)* **127**,**028.87** *(126*,*690.26–127*,*367.48)*	23,417.19 25,026.79	**17.97** **19.50**	0.53 0.64	**13.66** *(13.63-13*,*69)* **12.92** *(12.90-12.94)*	0.40 0.43	58,542.98 58,201.84	**1**,**252**,**269.29** *(1*,*249*,*677.10-1*,*254*,*861.48)* **1**,**164**,**961.36** *(1*,*162*,*711.58-1*,*167*,*211.14)*
Tirzepatide	**208**,**225.05** *(207*,*727.30–208*,*722.80)* **229**,**266.58** *(228*,*782.34–229*,*750.82)*	117,781.76 127,264.50	**18.33** **19.93**	0.89 1.07	**13.92** *(13.89–13.94)* **13.19** *(13.17–13.21)*	0.66 0.70	178,457.21 181,806.43	**1**,**183**,**530.29** *(1*,*181*,*136.78-1*,*185*,*923.80)* **1**,**089**,**593.15** *(1*,*087*,*541.90-1*,*091*,*644.39)*

### Results of sensitivity analysis

To examine uncertainty introduced by parameter variability, multiple one-way sensitivity analyses were performed and tornado diagrams, combining multiple one-way SAs, were computed.

Due to the complex and interdependent structure of transition probability formulas within this model only patient age, cost parameters and QoL values were able to be directly evaluated through sensitivity analysis.

**Fig. 3:**
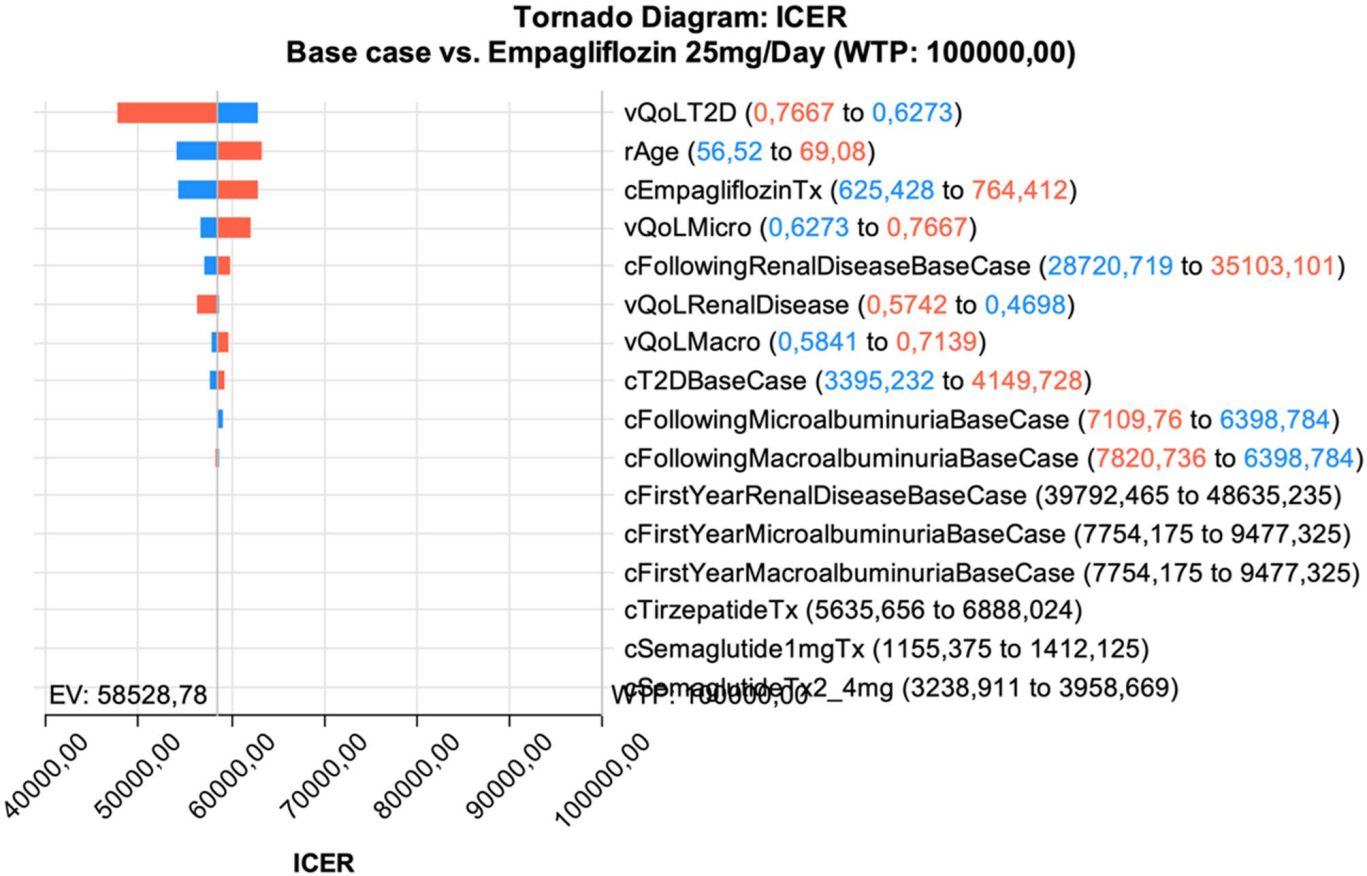
Tornado diagram comparing base case against Empagliflozin at a WTP threshold of 100,000€ for 62.8-year-old female patients

Figure 3 illustrates the results of a multiple one-way SA of base case against Empagliflozin with the listed variables being changed by ± 10%. QoL associated with the T2D health state proved to be the most influential factor affecting the incremental cost-effectiveness ratio. The second most impactful variable was the patient age followed by drug cost for Empagliflozin.

The tornado diagrams for other interventions against standard of care can be found in the supplementary material.

### Results of price threshold analysis

With Tirzepatide not being considered cost-effective at the list price, a price threshold analysis was performed for both age scenarios and separated by sex. To achieve the WTP threshold of 100,000€ Tirzepatide cost should be below 3,161.24€ per annum for men and below 3,236.22€ for women respectively. In the alternative analysis scenario with patients being 50 years old, treatment cost would have to be below 3,237.78€ for men and below 3,288.84€ for women.

## Discussion

This study provides a comparison of modern second line therapy added to standard care against first line therapy alone. Here, two GLP1 RA, namely Semaglutide and Tirzepatide, and the SGLT2 inhibitor Empagliflozin, all as second-line therapy, were compared against standard first-line T2D treatment alone to inspect preventative effects on kidney damage. All these comparators are licensed in Germany as add-ons to standard care [37] and they are known to show a measurable benefit for patients.

### Added research value

An increase in the life expectancy, regarding diabetes related kidney damage, in the range of 0.30 to 0.64 years for men and between 0.39 and 0.84 years for women mean significant improvement in survival from the patient’s perspective. It does not surprise that additional medical interventions lead to higher costs. In our analysis with a willingness-to-pay threshold of 100,000€/ QALY gained Semaglutide and Empagliflozin prove to be cost-effective.

Another important finding is that intervention in older patients does not mean lower cost-effectiveness despite their inevitably lower life expectancy. The results of this study are significant in light of the current discussion about triage in the German healthcare system due to limited resources [38].

A further contribution of this study is that it illustrates how improved survival can alter the observed incidence of renal disease. In our simulation the lowest annual incidence of renal disease occurs in the base case cohort (see Fig. 2) whereas all comparators show higher renal incidence over time. This is explained by competing risks, as all three comparators effectively prevent death by renal complications. More individuals survive long enough to enter the renal disease state, while in the base case a large proportion of these patients die earlier and therefore never develop renal disease.

An additional insight of this analysis is the exploration of parameter uncertainty for additional medical interventions. For all three comparators, health-related quality of life and drug price ranked amongst the most influential parameters. For Tirzepatide, the one-way SA indicates that uncertainty in these inputs exerts a relatively greater influence on the incremental cost-effectiveness than for Semaglutide and Empagliflozin. This can be explained by the combination of its higher acquisition cost and the fact that most of its incremental clinical benefit is captured through gains in QALYs.

### Limitations

This study relies solely on data input from scientific literature. Since a comprehensive Network-Meta-Analysis (NMA) reflecting the German healthcare context is currently lacking, any treatment comparisons presented in this study are derived from naïve direct comparisons. Consequently, there is additional uncertainty associated with the relative efficacy and cost-effectiveness differences observed between the three comparators. Nevertheless, this exploratory approach offers a high degree of transparency and reproducibility, as all model inputs are fully accessible to the public.

As baseline characteristics were sourced from the ACCORD trial conducted one decades ago, patient profiles and treatment standards over time may have changed to a degree of limiting the representativeness of the model population for current clinical practice.

Semaglutide in the dosage of 1 mg was launched specifically for the use in T2D patients. Semaglutide has also been brought to the market in a dosage of 2.4 mg but only for the use in obesity. Since obesity and T2D are closely connected to each other there is an unknown use of Semaglutide at 2.4 mg in obese patients also being diabetic. Due to no formal approval for the higher dosage in T2D patients, no literature is available at the time of this study investigating the effects on diabetic patients and related complications such as nephropathy.

No patient co-payments or rebates negotiated between manufacturers and sickness funds were taken into account when calculating intervention costs. Since the prices actually paid under the German statutory health care system are confidential under the German Medical Research Act (Medizinforschungsgesetz, 2024) [39] the listed prices for Tirzepatide and Semaglutide used in this model are likely to overestimate actual expenditures of the sickness funds. For the time being, health economic research from Germany is hampered seriously by the confidentiality of true drug prices.

Furthermore, prices for medication were taken for the reference year 2025 whereas state costs reference the year 2023. Price conversion for medication prices was not possible at the time of this study due to no data on health sector specific expenditures was available for 2025. However, this has no impact on the health economic implications, as drastic price changes within two years are unrealistic and all drug prices are treated equally for the chosen comparators.

Only diabetes medications were investigated in the prevention and slowing of the progression of nephropathy. Kidney-specific therapies, such as blood pressure-lowering drugs, were not considered in the advanced model states. However, this aspect is indirectly reflected in the use of kidney-specific risk equations. At the same time, the results of this study do not allow conclusions to be drawn about the extent of the independent effect of diabetes-specific versus kidney-specific therapy. Second-line diabetes medications have demonstrated significant cardioprotective benefits by reducing the risk of major cardiovascular events in diabetes patients. As this study exclusively focuses on kidney-specific outcomes, these cardiometabolic effects were not considered. Consequently, the overall cost-effectiveness of the investigated treatments is likely to be underestimated. The general cardiometabolic mortality was considered by its respective ICD codes in the used mortality table for Germany.

In this study we primarily relied on multiple one-way sensitivity analyses to explore the relation between individual model inputs and the key outcome parameters. Health economic guidance documents such as the ISPOR-SMDM Modeling Good Research Practices Task Force-6 [40] generally recommend the use of probabilistic sensitivity analyses (PSA). As we neither had access to individual patient data nor to statistical distributions, we decided to base the sensitivity analysis on the method that required the least data quality, hence one-way sensitivity analysis only. Nevertheless, we have performed PSA, and its results are available in the electronic supplementary material. PSA results were consistent with base-case and one-way sensitivity analyses results and do not change the overall health economic implications.

### Generalizability

Most of the medical evidence used in this study is derived from multinational trials, suggesting a high degree of generalizability for the clinical outcomes. However, the question of economic transferability arises with German cost data being used. Cost data cannot be seen globally and may vary significantly for different countries considering different health care systems and insurance policies.

The patients in this model were older individuals with preexisting risk factors that make them susceptible to renal complications. Consequently, the extend of generalizability for these results to younger or generally healthier patients remains uncertain. Second-line therapy may exert a greater preventive effect in lower-risk diabetes patients, but this potential effect modification could not fully be investigated in this study.

### Future research

Despite the robustness and consistency of the presented results of this study the need for more patient-centred clinical trials remains unbroken. Real-world evidence should be prioritized to confirm generalizability across different healthcare settings. This analysis should be repeated on large real-world datasets to provide more nuanced assessments of long-term effects.

It should be considered that this study is isolating the therapy effects on kidney disease. It would be desirable that future modelling studies include all other medical and cost aspects of T2D for instance, second-line therapeutic interventions also have cardioprotective effects [41] which make them overall more effective, yet to a health economically unknown extent.

## Conclusion

This study shows that the second-line therapies Empagliflozin, Semaglutide and Tirzepatide for type 2 diabetes patients achieve relevant medical improvements in the prevention and deceleration of nephropathy at higher expenditures for the German health system. While the interventions improve medical outcomes an overall cost-effectiveness is achieved in two out of three scenarios at the chosen WTP threshold. Age and sex did not prove to be relevant factors influencing the cost-effectiveness.

## Supplementary Information

Below is the link to the electronic supplementary material.

Supplementary Material 1

## Data Availability

A functional Excel Markov model copy has been developed to allow interested experts additional insight into this exploratory study. The model is available as additional electronic supplementary material.

## Abbreviations

T2D: Type 2 diabetes
eGFR: Estimated glomerular filtration rate
CHEERS: Consolidated Health Economic Evaluation Reporting Standard
LE: Life expectancy
QALY: Quality adjusted life years
ICUR: Incremental cost utility ratio
NMB: Net monetary benefit
WTP: Willingness to pay
QoL: Quality of life
HR: Hazard ratio
SA: Sensitivity analysis
PSA: Probabilistic sensitivity analysis
NMA: Network Meta Analysis
