# Supplementary material for: The cost-effectiveness of second line diabetes medication added to standard therapy in preventing type 2 diabetes related nephropathy in Germany
Source: Cost Eff Resour Alloc. 2026 Jul 27;24:96. doi: 10.1186/s12962-026-00801-5 (PMC13404957; doi:10.1186/s12962-026-00801-5)
Supplement: Supplementary file 1 — Supplementary Material 1 [file 12962_2026_801_MOESM1_ESM.docx]

# Supplementary Material Available for this Publication

A functional [Excel Markov model copy](https://www.hs-heilbronn.de/de/nephropathy-02d666e20bfdcdd1) has been developed to allow interesed experts additional insights into this exploratory study. Minimal deviations in the calculated results are explained by the deterministic Markov calculations in contrast tot he stochastic microsimulation used in the main TreeAge model.


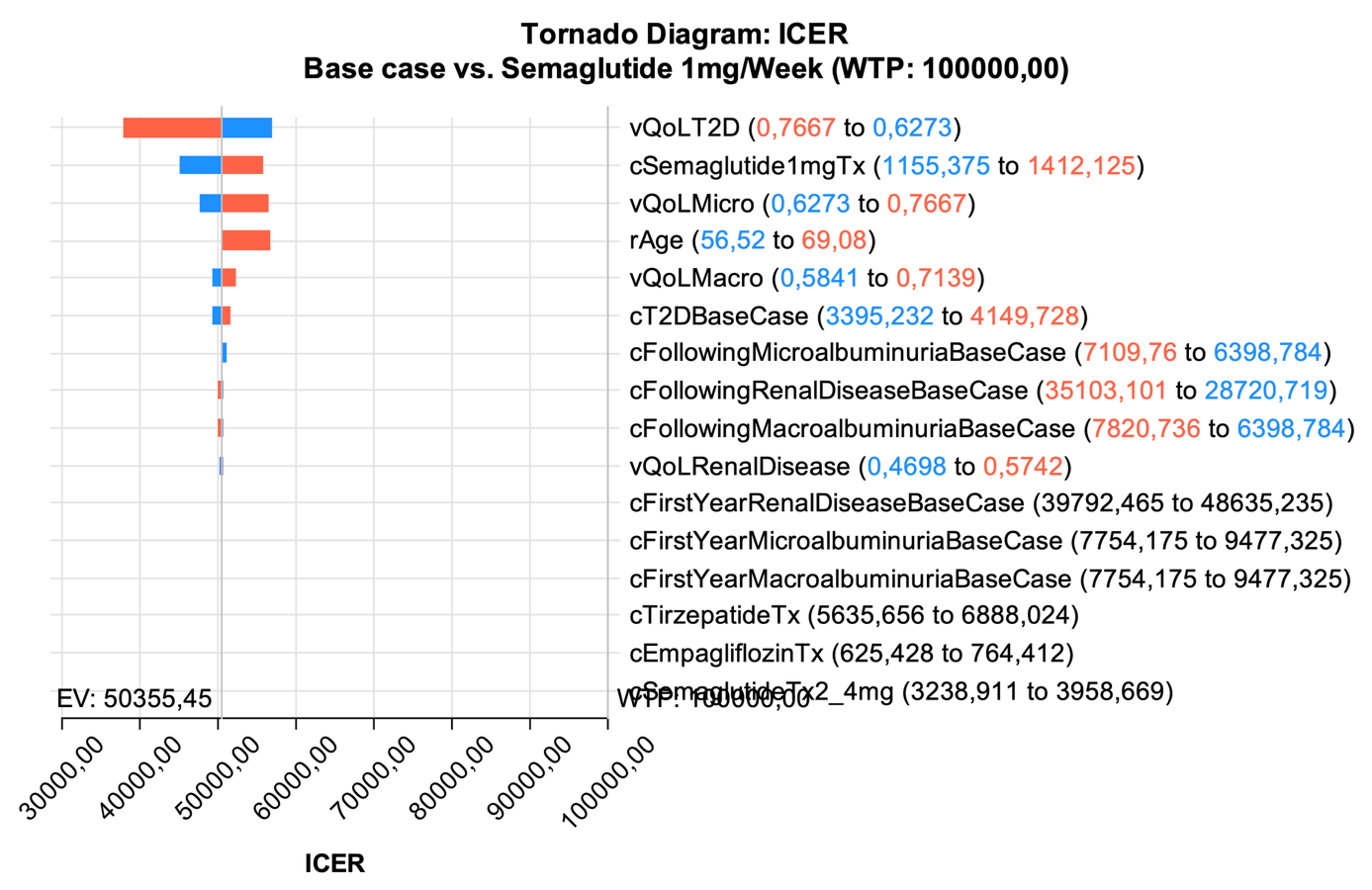


Figure 1: Tornado diagram comparing base case against Semaglutide at a WTP threshold of 100,000€ for 62.8-year-old female patients.


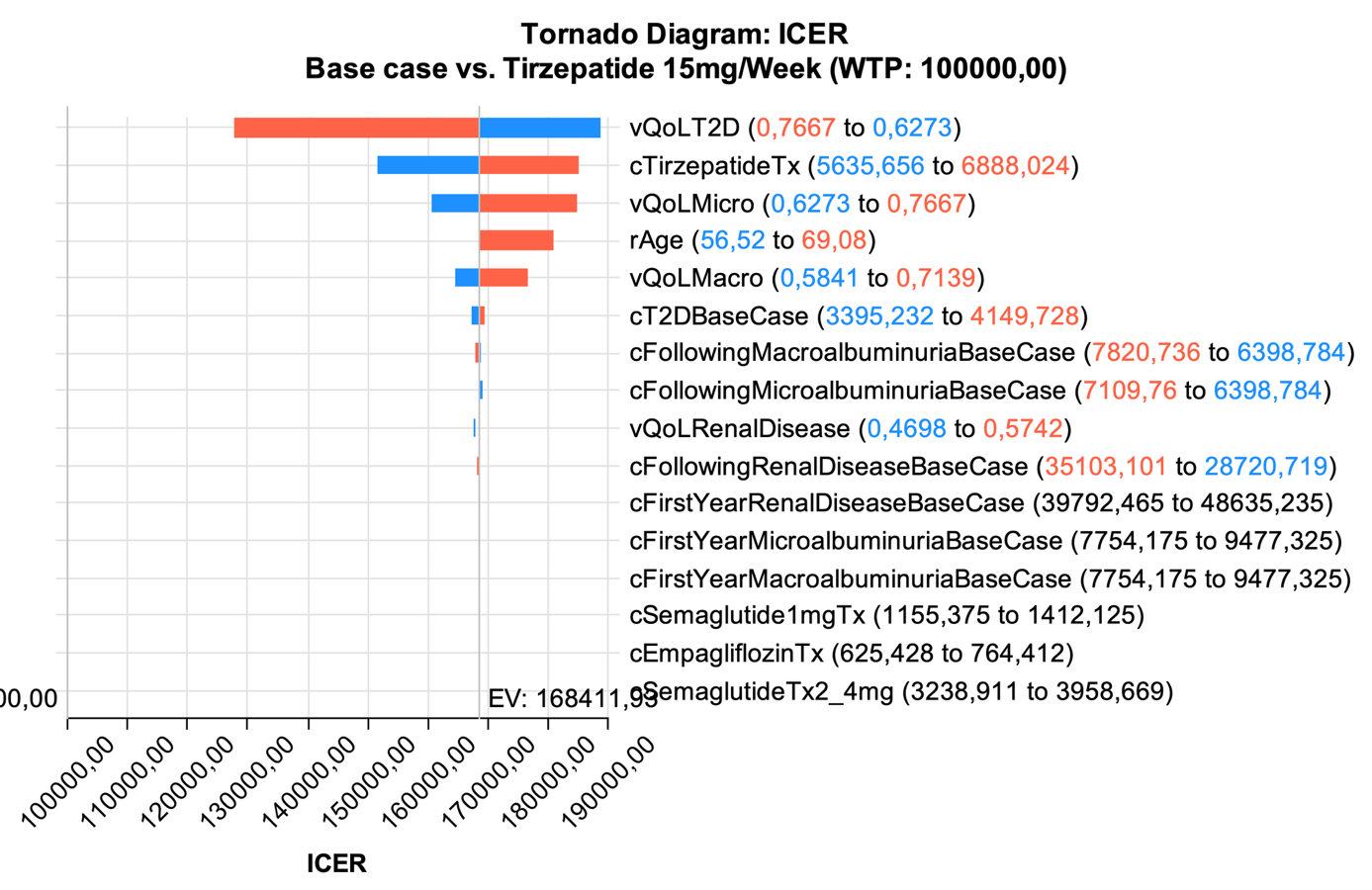


Figure 2: Tornado diagram comparing base case against Tirzepatide at a WTP threshold of 100,000€ for 62.8-year-old female patients.


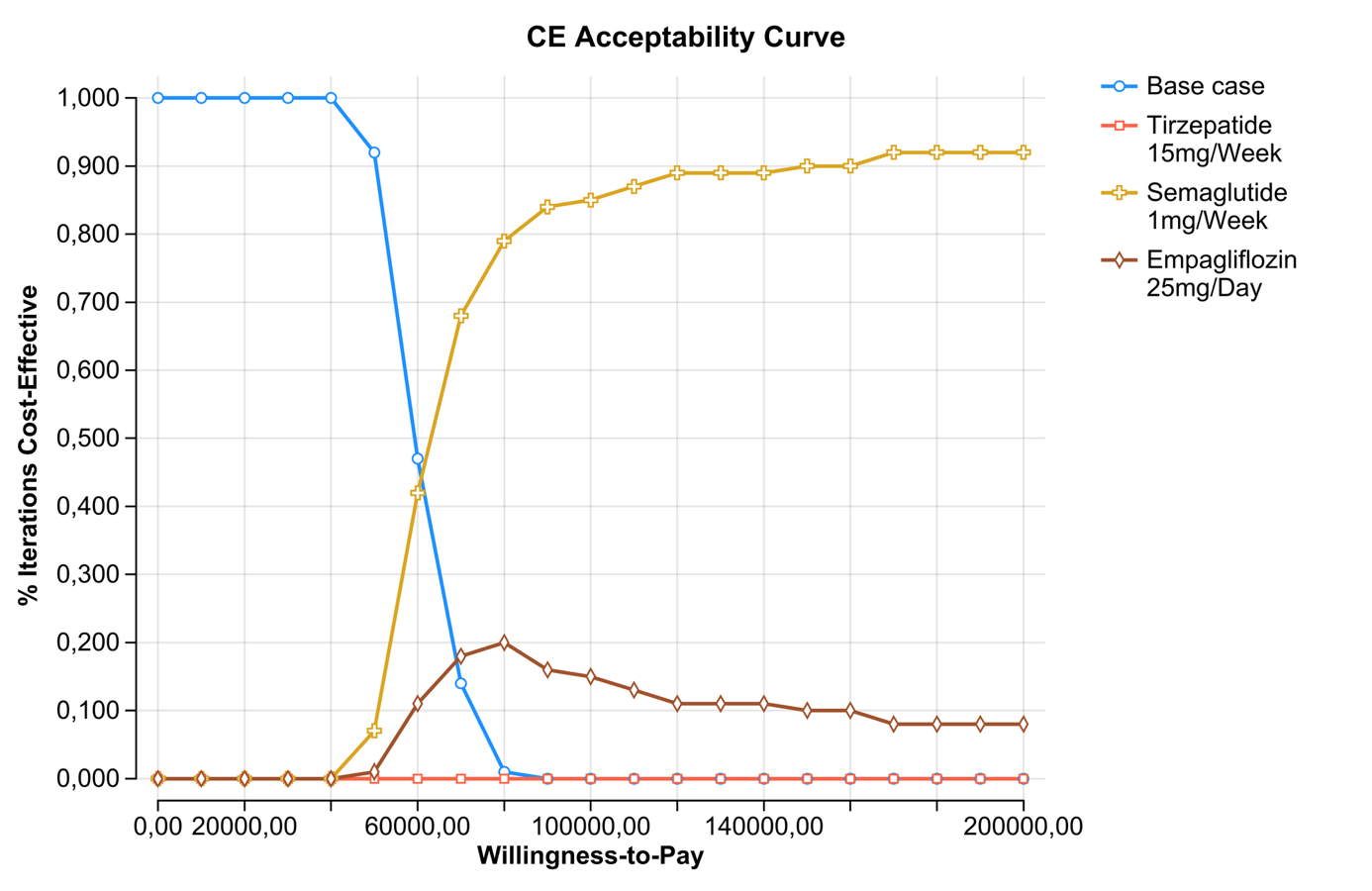


Figure 3: Cost-effectiveness acceptability curve for WTP thresholds until 200.000€.


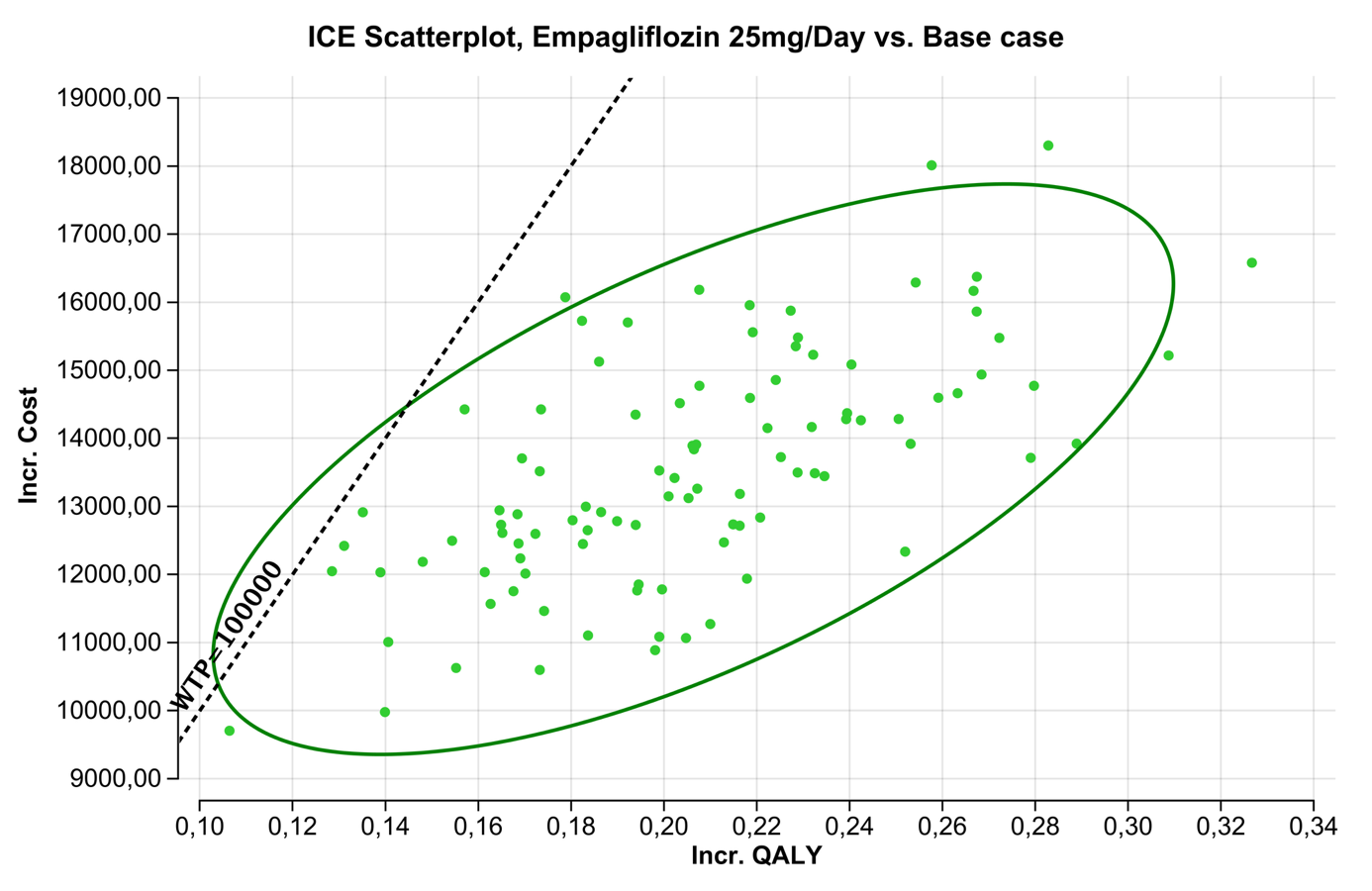


Figure 4: Incremental Cost-Effectiveness Scatterplot comparing Base Case vs. Empagliflozin treatment with a WTP threshold of 100.000€.


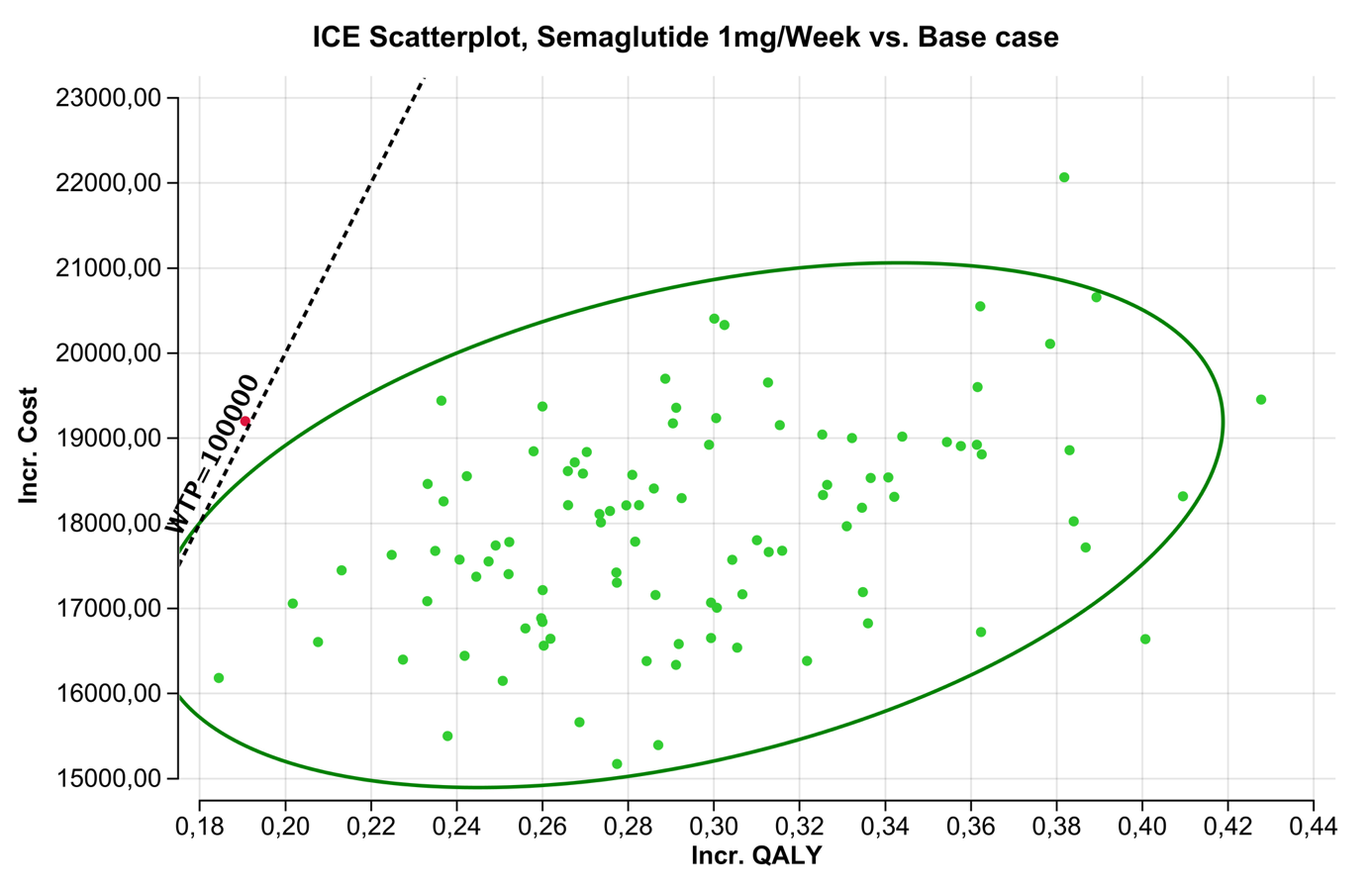


Figure 5:Incremental Cost-Effectiveness Scatterplot comparing Base Case vs. Semaglutide treatment with a WTP threshold of 100.000€.


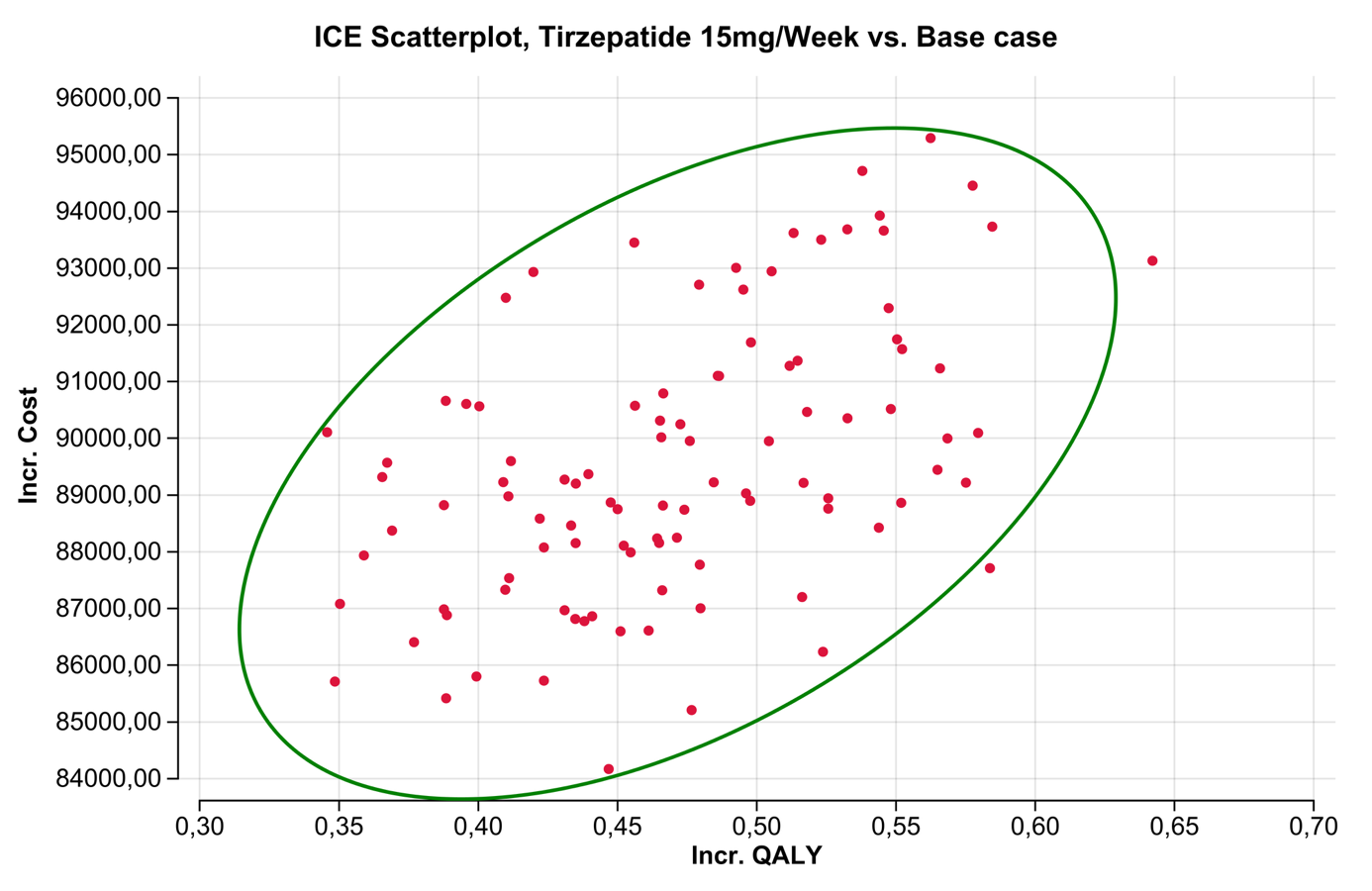


Figure 6: Incremental Cost-Effectiveness Scatterplot comparing Base Case vs. TIrzepatide treatment with a WTP threshold of 100.0

| Citation | Study abbreviation | Standard of care | Patient age (years) | HbA1c (%) | HDL  (mg/dL) | Total cholesterol (mg/dL) |
| --- | --- | --- | --- | --- | --- | --- |
| Ahrén B, Masmiquel L, Kumar H, Sargin M, Karsbøl JD, Jacobsen SH, Chow F. Efficacy and safety of once-weekly semaglutide versus once-daily sitagliptin as an add-on to metformin, thiazolidinediones, or both, in patients with type 2 diabetes (SUSTAIN 2): a 56-week, double-blind, phase 3a, randomised trial. Lancet Diabetes Endocrinol. 2017 May;5(5):341-354. | SUSTAIN 2 | Metformin, Thiazolidinediones, Metformin + Thiazolidinediones | 56 | 8.0 | --- | --- |
| Zinman B, Wanner C, Lachin John M, Fitchett D, Bluhmki E, Hantel S, et al. Empagliflozin, Cardiovascular Outcomes, and Mortality in Type 2 Diabetes. New England Journal of Medicine.373(22):2117-28. | EMPA-REG OUTCOME | Background glucose-lowering therapy remained unchanged. | 63.2 | 8.06 | 44.5 | 163.3 |
| Davies M, Færch L, Jeppesen OK, Pakseresht A, Pedersen SD, Perreault L, et al. Semaglutide 2·4 mg once a week in adults with overweight or obesity, and type 2 diabetes (STEP 2): a randomised, double-blind, double-dummy, placebo-controlled, phase 3 trial. Lancet. 2021;397(10278):971-84. | STEP 2 | Metformin, sulfonylureas, SGLT2 inhibitors, or thiazolidinediones | 56 | 8.1 | 42.54 | 174,02 |
| Ludvik B, Giorgino F, Jódar E, Frias JP, Fernández Landó L, Brown K, et al. Once-weekly tirzepatide versus once-daily insulin degludec as add-on to metformin with or without SGLT2 inhibitors in patients with type 2 diabetes (SURPASS-3): a randomised, open-label, parallel-group, phase 3 trial. The Lancet. 2021;398(10300):583-98. | SURPASS-3 | Background metformin alone or metformin and SGLT2 inhibitors | 57.5 | 8.21 | --- | --- |
| Emanuelsson F, Jensen J, Omar M, Jürgens M, Kistorp C, Brandt-Jacobsen NH, et al. Effect of empagliflozin on plasma lipids and lipoproteins in type 2 diabetes and heart failure - Empire HF and SIMPLE. J Clin Lipidol. 2025;19(2):276-85. | SIMPLE | Standard T2D care and lipid lowering treatment in 73.2% | 67.5 | 7.6 | --- | --- |
| Hiddo J.L. Heerspink, Naveed Sattar, Imre Pavo, Axel Haupt, Kevin L. Duffin, Zhengyu Yang, Russell J. Wiese, Jonathan M. Wilson, Andrea Hemmingway, David Z.I. Cherney, Katherine R. Tuttle; Effects of Tirzepatide Versus Insulin Glargine on Cystatin C–Based Kidney Function: A SURPASS-4 Post Hoc Analysis. Diabetes Care 1 August 2023; 46 (8): 1501–1506. | SURPASS-4 | Combination of metformin, sulfonylurea agent, or sodium–glucose cotransporter-2 inhibitors | 63.0 | 8.5 | --- |  |
| Perkovic V, Tuttle Katherine R, Rossing P, Mahaffey Kenneth W, Mann Johannes FE, Bakris G, et al. Effects of Semaglutide on Chronic Kidney Disease in Patients with Type 2 Diabetes. New England Journal of Medicine. 2024;391(2):109-21. | FLOW | RAS inhibitors (angiotensin-convert-  ing–enzyme inhibitor or angiotensin-receptor  blocker). | 66.6 | 7.8 | --- | --- |
| Wanner C, Inzucchi SE, Lachin JM, Fitchett D, Eynatten Mv, Mattheus M, et al. Empagliflozin and Progression of Kidney Disease in Type 2 Diabetes. New England Journal of Medicine. 2016;375(4):323-34. | EMPA-REG OUTCOME | Background glucose-lowering therapy remained unchanged. | 67.1 | 8.07 | 44.2 | --- |

Figure 7: Overview over trials use to estimate treatment effects.
